# Investigation of Differences in P53 Gene Polymorphisms between Schizophrenia and Lung Cancer Patients in the Turkish Population

**DOI:** 10.4061/2011/483851

**Published:** 2011-03-03

**Authors:** Ulku Özbey, Hüseyin Yüce, Mustafa Namli, Tamer Elkiran

**Affiliations:** ^1^Genetic Department, Faculty of Veterinary Medicine, Yüzünci Yil University, Zeve Campus, 65080 Van, Turkey; ^2^Department of Medical Biology and Genetics, Faculty of Medicine, Firat University, 23119 Elazig, Turkey; ^3^Psychiatry Hospital, 23200 Elazig, Turkey

## Abstract

*Objective*. The reduced incidence of cancer observed in schizophrenia patients may be related to differences in genetic background. It has been suggested that genetic predisposition towards schizophrenia is associated with reduced vulnerability to lung cancer, and p53 gene is one of the candidate genes. In our study, we aimed to investigate polymorphisms in the BstUI in exon 4 and MspI in intron 6 restriction sites of the p53 gene in Turkish schizophrenia patients, lung cancer patients, and controls. *Material and Methods*. Allele and genotype incidence of these polymorphisms with their haplotype combinations were studied in 100 Turkish lung cancer and schizophrenia patients and 100 controls without malignant and schizophrenia diseases. The genotype characteristics were determined by PCR-based RFLP method using DNA extracted from peripheral blood. *Results*. For the BstUI and MspI polymorphism, there were found significant differences in the genotype and allele frequencies between schizophrenia and lung cancer patients with control groups (*P* < .01). The analysis based on haplotype frequencies showed the presence of BstUI-MspI 2-1 haplotype in cancer patients (12%) in contrast to the absence of this haplotype in schizophrenia and controls. Only in lung cancer patients we found both significant decrease of A1 allele of the p53 codon 72 (OR 0.23, 95% CI 0.9–0.58) and A1/A1 homozygous genotype (*P* < .0001, OR 0.19). *Conclusion*. The results of this study suggest a protective effect of A1 allele against lung cancer, and the p53 MspI polymorphism may modify the susceptibility to lung cancer as a single factor rather than in combination with BstUI polymorphism.

## 1. Introduction

The relationship between schizophrenia and cancer has posed an epidemiological puzzle for decades [1]. Lower incidenceof malignancies, especially lung cancer, in patients of schizophrenia compared to the general population has been reported in several studies [2, 3]. Recently, reduced risk of cancer in patients of schizophrenia has been reported in an additional study. Theories proposed to date attempting to explain the reduced risk of cancer in schizophrenia patients focused on environmental, genetic, pharmacological, and psychosomatic factors [4]. Particular attention has been paid to the question whether genetic predisposition towards schizophrenia confers genetically reduced susceptibility to cancer. The protein product of the tumor suppressor gene p53 is a cell-cycle checkpoint control protein that assesses DNA damage and acts as a transcription factor regulating genes, which control cell growth, DNA repair, and apoptosis. However, little is known whether patients with schizophrenia possess genetic factors that also confer tumor resistance, especially to lung cancer [5, 6], which suggested that the reduced incidence of cancer observed in schizophrenia patients might be linked to differences in apoptosis and proposed p53, a tumor-suppressor gene, which is considered as a candidate gene for the susceptibility. The p53 gene is one of the most frequently mutated genes in all types of cancers including lung cancer [6]. 

P53 variable number of tandem repeats- (VNTRs-) and single-nucleotide polymorphisms- (SNPs-) based approaches have previously been employed to determination of possible individual susceptibility and association of these polymorphic sites within p53 gene with lung cancer [6]. Two polymorphic sites in the sequence of the p53 gene, a BstUI restriction site at codon 72 in exon 4 and an MspI site in intron 6, have been proposed as responsible for the susceptibility to lung cancer [7, 8]. In this study, we investigated polymorphisms in the two restriction sites of the p53 gene in Turkish schizophrenia patients, lung cancer patients, and controls.

## 2. Material and Methods

### 2.1. Subjects

In our hospital-based case-control study, 100 schizophrenic patients (54 males and 46 females), 100 lung cancer patients (90 males and 10 females), and 100 controls (59 males and 41 females) without schizophrenia were recruited. The schizophrenia patient group consisted of 100 Turkish schizophrenia patients diagnosed based on DSM-IV; mean age was 37.2 ± 10.0 years (mean ± SD). The lung cancer patient group consisted of 100 Turkish patients diagnosed with histological proven diagnosis of lung cancer; the mean age was 60.7±10.8 years. The 100 normal controls without schizophrenia and lung cancer diseases was recruited; the mean age was 50.3 ± 17.4 years (mean ± SD). The control group consisted of unrelated healthy volunteers free from present, past, and family history (first-degree relatives) of psychiatric illness and any cancer histories or substance abuse and the exclusion criteria for the patients. The group consisted of medical students and hospital personnel of Firat University Medical School. Subjects with the above-mentioned concomitant diseases were excluded from the study after relevant laboratory tests and consultations with appropriate physicians. Control group was age adjusted according to patients. After the complete description of the study to the subjects, all subjects gave informed written consent approval was obtained from the local Ethic Committee (Ethic Committee of the Firat University Medical Faculty).

### 2.2. DNA Extraction

DNA was prepared from 1 to 5 mL blood samples according to salting-out method [7, 9]. The DNA samples were then stored at 4°C until used as a template DNA in polymerase chain reaction (PCR).

### 2.3. Genotyping

**Figure 1:**
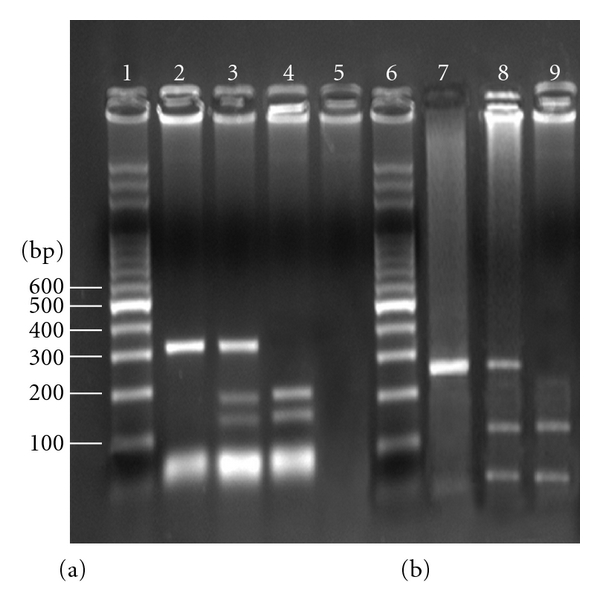
Agaros gel electrophoresis of the p53 BstUI and the p53 MspI PCR products digested with BstUI and MspI restriction enzymes. (a) A1 allele does not create BstUI restriction site. The PCR product has 318 bp. Presence of CGCC sequence in A2 allele creates BstUI restriction site which leads to two fragments 182 bp and 136 bp. (b) A1 allele does not create MspI restriction site. The PCR product has 240 bp. Presence of CCGG sequence in A2 allele creates MspI restriction site which leads to two fragments 164 bp and 76 bp. Lanes 1, 6: 100 bp DNA ladder; lanes 2, 7: 1-1 homozygotes; lanes 3, 8: 1-2 heterozygotes; lanes 4, 9: 2-2 homozygotes; lane 5: no DNA in PCR reaction (blank).

DNA was prepared from 1 to 5 mL blood samples according to salting-out method [9]. Genotyping of the two polymorphisms, the exon 4 BstUI (codon 72), and the intron 6 MspI RFLPs were performed as described previously [7]. Briefly, polymerase chain reaction (PCR) amplification of two fragments encompassing the p53 BstUI RFLP and the MspI RFLP was performed in total volume of 50 ml containing 250 ng of genomic DNA, 1.5 mM MgCl_2_, 100 mM dNTP, 15 pmol of each primer, and 0.5 U Taq polymerase (GibcoBRL®). PCR involved an initial denaturation at 94°C for 5 minutes, followed by 35 cycles of denaturation at 94°C for 1 minute, annealing at 58°C for 1 minute, and extension at 72°C for 1 minute, with a final extension at 72°C for 12 minutes. The BstUI RFLP sense primer 5′-TTT CAC CCA TCT ACA gTC CC-3′ and the antisense primer 5′-ACC TAg GCT CAg ggC AAC TgA CCg-3′ amplified a 318-bp PCR product [10]. The MspI RFLP sense primer 5′- TAT gAg CCg CCT gAg gTC Tgg-3′ and the antisense primer 5′-TAC Agg CAT gAg CCA CTg CgC-3′ amplified a 240-bp PCR product [11]. Ten percent of PCR products were digested with BstUI (New England BioLabs) at 60°C for 2 h with MspI (New England BioLabs) at 37°C for 3 h. Digests were separated on a 3% agarose gel with ethidium bromide. The electrophoresis was run for 2.5 h at 150 V. DNA fragments were stained with ethidium bromide. The ethidium bromide staining fragments were analyzed on UV source using the image analysis system Kodak EDAS 120 (Figure 1).

In this study we inspected two polymorphic sites within the sequence of, the p53 gene: a BstUI restriction fragment length polymorphism (RFLP) at codon 72 in exon 4 and a MspI RFLP in intron 6. The BstUI RFLP recognizes a SNP at codon 72 that either encodes a proline (CCC, allele A1) or an arginine (CGC, allele A2). MspI recognizes polymorphic site in intron 6 consisting of either six or eight variable bases. The BstUI A1 and the MspI A1 allele are both alleles not presenting the restriction enzyme recognition sites. The presence of each restriction site was indicated as “A1”; “A2” was used to indicate absence of the restriction site. Subjects were scored as “A1–A1”, “A2–A2” (homozygotes), or “A1–A2” (heterozygote) according to the restriction pattern [12]. 

### 2.4. Statistical Analyses

Genotype allele and haplotype frequencies of schizophrenia and lung cancer patients with control groups were compared via the chi-square test and Fisher's exact test. Comparisons for sex, genotype, and allele frequencies for lung cancer patients, schizophrenic patients, and healthy controls were performed using the chi-square test. The odds ratio (OR) and its 95% confidence interval (95% CI) were used to analyze the frequencies of alleles and genotypes. For all of the tests, the criterion for significance was set at *P* < .05. Data are presented as mean ± SD. Hardy-Weinberg equilibrium was tested for with a goodness of fit *x* ^2^ test with 1 degree of freedom to compare the observed genotype frequencies among the subjects with the expected genotype frequencies. All calculations were performed with the SPSS (Statistical Packages of Social Sciences, SPSS for Windows, Version 11.5, Inc., Chicago, IC, USA) 15.0 statistical package.

## 3. Results

The characteristics of the study groups are illustrated in Table 1. Healthy controls (100 individuals) were about similar age as lung cancer patients (100 individuals). The male/female distribution in the healthy group was 59% versus 41%, in the lung cancer group 90% versus 10%, and the schizophrenia group 54% versus 46%. Smokers were 43% in the control group and 88% in the lung cancer group and 94% in the schizophrenia group. Alcohol consumption were 12% in the control group, 34% in the lung cancer group, and 30% in the schizophrenia group (Table 1). The most abundant type of cancer determined histologically was squamous cell cancer (32%), followed by adenocarcinoma (16%), large cell carcinoma (9%), and small cell carcinoma 29%. However, there was an important group without histological determination (*n* = 14, 14%).

The genotype frequencies of BstUI and MspI RFLPs in the schizophrenia, lung cancer, and control groups are shown in Table 2. For the BstUI polymorphism, there was found significant difference in the genotype frequencies between the control and the lung cancer patient groups (genotype frequency: *P* < .001). However, the genotype of the MspI A1A2 showed significant differences between the schizophrenia patient and the control groups (OR 0.81, %95 CI 0.64–1.1, *P* < .001). In addition, the proportion of genotype of the MspI A1A2 was increased in the schizophrenia (54%) in comparison to the lung cancer (44%). However, this deviation did not reach a statistical significance (*P* = .424). The frequency of A2A2 genotype among lung cancer and schizophrenia patients was identical (46%) (Table 2).

The allele frequencies of BstUI and MspI RFLPs in the schizophrenia, lung cancer and control groups are shown in Table 3. In allele frequencies of BstUI and MspI RFLPs, significant allele frequency differences were found between patients and the controls (*P* < .001). In our case-control study, a decreased frequency of the p53 BstUIA1 allele was found in lung cancer patients (*P* = .09, OR 0.23, 95% CI 0.9–0.58). However, a significant increase of the BstUI A2 and MspI A1 allele frequency was found in lung cancer patients (OR 1.22, %95 CI 1.09–1.036; OR 0.87, %95 CI 0.81–0.94) (Table 3).

The allelic or genotype frequencies based on two p53 polymorphisms were estimated in male and female in patients and controls. In lung cancer, schizophrenia patients and Turkish healthy controls stratified by sex and smoking, and significant differences were observed in genotype frequencies (*P* < .01). In the nonsmoker patients groups and controls no significant differences were observed in the genotype frequencies (*P* = .201). 

On comparing control and lung cancer patients, groups stratified by smoking, it is possible to observe that only smokers differ significantly in allelic frequencies (*P* < .001). In the non-smoker schizophrenia and lung cancer no significant differences were observed in the allelic frequencies. The allelic frequency BstUI A1 was higher in male smokers with lung cancer (1.430) than in the control group (0.416). The non-smoker group does not present differences between controls and lung cancer patients (*P* = .376). The analyses in male non-smokers (both control and cancer groups) showed no differences in the allelic or genotype frequencies between both groups, suggesting the participation of this p53 polymorphism in lung cancer associated with smoking. The frequency of the p53 BstUIA1 allele among schizophrenia patients (males and females) was increased in the smoker males and female in comparison to nonsmoker males and females. Statistical association was demonstrated for allele frequency in schizophrenia patients (males and females) when the sample population was stratified according to sex and smoking (*P* < .01).

Risk of lung cancer from BstUI and MspI genotype with reference to histological types are shown in Tables 4(a) and 4(b). After grouping of patients according to four main histological types of lung cancer (epidermoid carcinoma, small cell carcinoma, large cell carcinoma, and adenocarcinoma), BstUI A1A1 genotype was detected, and adenocarcinoma (40%) in the lung carcinoma population was increased in the other histologies. In several epidemiological studies, there was association between BstUI A1A1genotype and adenocarcinoma risk. Furthermore, we observed a slight increase of BstUI A2/A2 genotypes frequency in patients with epidermoid and small cell carcinoma. The A1A1 genotype may also play a role in four main histological types of lung cancer development (Tables 4(a) and 4(b)).

We found a significantly higher proportion of p53 MspI heterozygotes (A1A2) in epidermoid and small cell carcinoma patients (*X* ^2^: 1.146, *P* = .979). Only the MspI heterozygotes (A1A2) that had a small cell carcinoma and epidermoid showed the significant difference from healthy controls (Tables 4(a) and 4(b)). OR was calculated to be 3.24 (95% CI 1.07–9.85, *P* = .979). The MspI heterozygotes with other histologies also showed the tendency towards increased OR, but this was not significant. Thus, there seems to be no consistent association between MspI heterozygous genotype and histological types of lung cancers.

The differences of allele frequencies were estimated according to histological types of lung cancer patients. The proportion of Pro BstUI and MspI (A1) allele was found in epidermoid carcinoma. Furthermore, we observed a slight decrease of BstUI and MsPI allele frequency in patients with adenocarcinoma and large cell carcinoma (Table 5).

The haplotype frequencies based on two p53 polymorphisms were estimated in patients (Table 6). When the haplotypes had been taken into account, although the distribution of haplotypes in lung cancer patients was similar to controls, we did find found an interesting phenomenon. The haplotype carrying the BstUI A2 allele and MspI A1 allele was not found in healthy controls and schizophrenia patients in contrast to the presence of this haplotype in lung cancer patients (12%). In addition, the estimated frequency of the BstUI A1- MspI A2 haplotype of schizophrenia patients (39%) was sixfold higher than that of lung cancer patients (6%). The BstUI A2-MspI A2 haplotype was observed in lung cancer patients (61%), which was detected in schizophrenia patients (82.2%). Thus, it could be suggested that the MspI A2 allele, by itself or perhaps more important in the form of the BstUI A1-MspI A2 haplotype, is a marker of susceptibility of lung cancer.

Table 7 presents an estimation of the haplotype frequencies in male and females in patients and the control groups. Sex differences were not found in the distribution of BstUI-MspI haplotype in common population. No significant (*P* = .844) differences were found in patients from the control group. The haplotype combination BstUI A2-MspI A1 among lung cancer patients (males and females) was increased in the males (12%) in comparison to females (10%), which was not detected in schizophrenia patients and control groups. However, this deviation did not reach statistical significance (*P* = .098) (Table 7).

## 4. Discussion

Lung cancer incidence is higher among women than men. Furthermore, replication would support the hypothesis by Henschke et al. that women may be more susceptible to develop lung cancer than men but are less likely to die from the disease [13]. Studies are also needed to determine whether the survival difference between male and female never smokers is large enough to account for the lower lung. Lung cancer is the most preventable of all of the major forms of cancer because 85% to 90% of deaths from lung cancer are a result of active cigarette smoking. Environmental tobacco smoke is often stated to be the major cause of lung cancer [14]. 

The majority of the causative factors of lung cancer are well established. Tobacco smoking is the most important factor. The incidence of lung cancer is related to the prevalence of smoking in a population. In a study from Denmark, it was observed that the incidence of lung cancer declined slightly in association with a decline in the prevalence of smoking in men [15]. Tobacco smoking continues to be a major problem in Turkey, where the number of smokers increases daily. In our study, we observed that approximately 90% of the patients were smokers or exsmokers. Smoking among women in Turkey is not as common as among men, and this was reflected in the predominance of male patients (90.4%) in this study. The dominance of males among lung cancer patients has also been observed in other studies from Turkey [16, 17]. 

This study shows that the frequency of epidermoid carcinoma was clearly higher than that of other subtypes. Epidermoid carcinoma is the most common subtype in Asian countries such as Korea and China [18, 19]. In contrast, adenocarcinoma is currently the most frequent histological type in the United States and Japan [20, 21]. Although adenocarcinoma is increasing in prevalence among European women, it is not yet the most predominant cancer in Europe [22]. The reasons for this shift are not clear although the rise in the incidence of lung cancer in women plays some part. Other possible factors include changes in smoking habits and cigarettes or other environmental exposure. Smokers of low-yield filter-tipped cigarettes have to take more frequent and larger puffs to satisfy their need for nicotine. This allows the cigarette smoke to reach the distant branches of the bronchioalveolar tree, where adenocarcinoma usually occurs [23]. In addition to these factors, some authors claim that the increase in the incidence of adenocarcinoma might be related to a better histological diagnosis of tumors which were previously called ‘unspecified epithelial carcinomas [24]. In our study, it is of interest to note that 16% of patients did not have a specific histological diagnosis, and 9% of patients were diagnosed as unspecified large cell carcinoma. The incidence of adenocarcinoma might, therefore, have been higher if the definitive histological subtypes of these patients had been diagnosed. Large cell carcinoma was observed at a low frequency in our study (9%) when compared with other studies from different countries (about 10% of lung cancers) [15, 24]. We cannot exactly explain the reason for this, but we think that some large cell carcinomas might have been established as unspecified large cell carcinoma due to the difficulty of making a diagnosis of large cell cancer in small, poorly preserved bronchoscopic biopsy specimens. Small cell carcinoma constitutes 20%–25% of lung cancer patients [15, 25]. We observed a similar frequency of small cell carcinoma (29%). This suggests that large cell carcinoma is diagnosed at more advanced stages in Turkey than in developed countries. The largest number of data so far collected in Turkey shows that the vast majority of patients with lung cancer are male, epidermoid cell is the most common histologicaltype, approximately 90% of patients are current or exsmokers, and only a small proportion of patients are diagnosed at an early stage. The difficulty in making an early diagnosis and in starting early treatment of lung cancer indicates the importance of combating tobacco. Education regarding the prevention of smoking initiation in youth and more effective efforts toward smoking cessation must be the main targets of the fight against lung cancer.

Cancer risk in schizophrenia patients has been discussed over decades. Several studies have reported reduced cancer incidence in schizophrenia patients in epidemiological perspectives [26]. But the mechanism underlying this different occurrence between cancer and schizophrenia has not been elucidated. Recently, genetic backgrounds, which regulate the various biological processes, are suggested as possible causes of this phenomenon. However, to date, few studies have been performed concerning the relationship between schizophrenia pathogenesis and cancer development. Previous studies have found that the incidence of cancer in schizophrenic patients is reduced. Especially, the incidence of lung cancer for schizophrenic patients is lower than for the general population, which is unexpected because schizophrenic patients smoke more than the general population. Secondly, schizophrenia is generally considered to be a neurodevelopmental disorder [27]. Candidate neurodevelopmental molecules related to cell proliferation, axonal outgrowth, synaptic regression, myelination, and cell migration might be implicated in schizophrenia [28]. Komarova et al. found that p53 was strongly expressed in specific mouse brain regions, suggesting that the p53 gene is involved in the development of the nervous system [27, 29]. Finally, there is evidence that increased expression of p53 in an early stage of brain development results in neuronal damage, and the increased neuronal apoptosis may partially explain the high incidence of neuromotor anomalies found in schizophrenia. The above findings point to the p53 gene as possibly being involved in the pathogenesis of schizophrenia.

When the distribution of genotype and allele had been taken into account, in our previous case-control study, a decreased frequency of the p53 MspI A1/A2 genotype was found in bladder cancer. More recently, both Wang et al. [29] and Kawajiri et al. [7] in earlier studies reported a relationship between the p53 BstUI A1/A1 genotype and lung cancer in Asiatic populations of Taiwan and Japan. In studies from Sweden of nasopharyngeal cancer and Germany of breast cancer, significant associations of the p53 BstUI A1/A1 and the p53 MspI A1/A2 genotypes were observed [8, 10, 30, 31]. The consistent association between the p53 SNPs (p53 BstUI and p53 MspI) and various forms of human malignancies may serve as arguments in favor of these polymorphic sites as genetic determinants of susceptibility. 

In our studies, significant difference was found in genotype and allele frequencies of the MspI and BstUI RFLPs between lung cancer patients, schizophrenia patients, and healthy normal Turkish control (data not shown). There was significantly higher BstUI A2A2 genotype in lung cancer patients (OR 0.73; OR 0.28). The frequencies of BstUI A1A1 genotype was found significantly lower (5%). In addition, control (22%) and patients with schizophrenia (38%) were observed higher of frequencies of BstUI A2A2 genotype (OR 0.19; OR 0.81). The Pro-Pro (BstUI A1A1, encoded proline) genotype was estimated to be associated with increased risk for lung cancer. The increased frequency of the *BstUl *Al (pro) allele was more pronounced in highly differentiated lung cancer. These results suggest that the *p53 BstUl *Al (pro) allele may influence increase lung cancer cancer. 

In our case-control study, a increased frequency of the p53 MspI A1/A2 genotype was found between the schizophrenia and the lung cancer patient groups. However, this deviation did not reach a statistical significance (*P* = .424). However, the significant differences in the genotype and allele frequencies of the MspI polymorphism in the p53 gene between the schizophrenia and the lung cancer patient groups suggest that the p53 polymorphisms could be a marker for lower susceptibility to lung cancer genetically in schizophrenia patients. On the other hand, there were some additional interesting results revealed. For the BstUI and MspI polymorphism, there was significant difference in the genotype and allele frequencies between the schizophrenia patient and the lung cancer patient groups (*X* ^2^ = 32.092*P* < .001; *X* ^2^ = 27.178, *P* < .001, Table 2 versus Table 3). In addition, a correlation between the presence of the MspI A2 allele and increased odds ratio for lung cancer (OR 2.98, 95% CI 1.46–6.09) was observed. The proportion of BstUI A1 allele was decreased in lung cancer patient in comparison to the healthy control and the schizophrenia. However, the incidence of BstUI A2 allele was increased in lung cancer patient. This results suggested that A1 allele could be a protective effect against lung cancer. Although incidence of A1 allele was observed slightly lower in lung cancer patient, the ratio of this allele was significantly increased. The above findings point to the A1 allele as possibly being involved in the pathogenesis of schizophrenia. Finally, this finding may explanation reason of the incidence of cancer in schizophrenic patients is reduced. This finding is important. Our results imply that genetic predisposition towards schizophrenia may offer reduced vulnerability to lung cancer.

The polymorphism of p53 gene at codon 72 consisting of either arginine- (Arg-) or proline- (Pro-) encoded allele is suggested to be associated with the susceptibility of tobacco-related lung cancer [32]. Yet, the BstUI A1 (Pro) allele carriers among the smoker lung cancer patients showed higher rate than control group. Thus, the present data showed that the p53 polymorphism could be associated with the susceptibility of tobacco-related lung cancer.

When the differences of genotype and allele frequencies estimated according to histological types of lung cancer had been taken into account, Fan et al. and Weston et al. [31, 32] reported that he *Pro/Pro *homozygous genotype occurred more frequently in adenocarcinomas. The prevalence of the *Pro/Pro *genotype in adenocarcinoma was higher than that of other genotypes and increased with increasing pack years. The prevalence of the *Pro/Pro *genotype in adenocarcinoma cases was statistically different from that of the controls. A Japanese study reported that the prevalence of the *Pro/Pro *variant in patients with adenocarcinoma was 1.2-fold higher, which was not statistically different from controls. [8] Weston et al. [32] reported a prevalence of 26% for their pooled control group and 21% in all types of lung cancer combined. Jin et al. [33] reported that the susceptible *Pro/Pro *genotype was associated with a 1.6-fold higher risk of all types of lung cancer combined in African Americans and 1.9-fold higher risk in Mexican Americans, with neither reaching statistical significance. In our study, the frequency of the *Pro/Pro *genotype in adenocarcinoma was much lower than that of the *Arg/Arg* genotype.

We found a significantly higher proportion of p53 MspI heterozygotes (A1 A2) in epidermoid and adenocarcinoma patients (*X* ^2^: 1.146, *P* = .979). However, among lung cancer patients who had p53 MspI heterozygous genotype, epidermoid and small cell carcinoma patients showed a significant difference (*P* = .979) when comparisons were based on histological types of lung cancer. Thus, there seems to be no consistent association between MspI heterozygous genotype and histological types of lung cancer. The fact that MspI polymorphism is located in noncoding region of the p53 gene (intron 6) offers support to the idea that this polymorphic site is not functionally involved in lung carcinogenesis but rather in linkage disequilibrium with some specific susceptibility site on the p53 gene. In addition to the BstUI A1A1 genotype of lung cancer patients (40%), adenocarcinoma and other histological types of lung cancer were detected. Several epidemiological studies have found an association between risk of adenocarcinoma and presence of BstUI A1A1 genotype. Interestingly, proportion of BstUI A2/A2 genotype was significantly higher in epidermoid carcinoma. The A1A1 genotype may influence the development of histological subtypes of lung cancer (Tables 4(a) and 4(b)). The Pro-Pro (BstUI A1A1, encoded proline) genotype was estimated to be associated with increased risk for lung cancer [6] because the apoptotic effect is lower than *Arg/Arg* genotype of this genotype. Epidemiological studies of genes that oppose and inhibit cell-cycle progression may shed additional light on the relationship between incidence of cancer and that of schizophrenia, the present authors believe that the literature is sufficiently consistent to suggest that the mechanism underlying the apparent tumour resistance in schizophrenia may be of aetiological significance to schizophrenia itself. One possible mechanism which plays a central role in both tumorigenesis and neurodevelopment is apoptosis. Apoptosis is the physiological process of genetically programmed cell death. Apoptosis is closely related to the processes promoting the life cycle of the cell. All cells contain clock-like machinery which controls orderly progression of the cell through the stages of its life cycle. These transitions start with mitosis (M) and proceed to a period of growth or differentiation (G1), hence to DNA synthesis (S) before a second period of growth (G2) leads again to mitosis, completing the life cycle. Cells that do not go on to mitosis may enter a quiescent growth-stage end point (G0). Growth factors and activated oncogenes are positive regulators of cell-cycle progression. Some genes, called tumour-suppressor genes, oppose cell-cycle progression and inhibit uncontrolled cell proliferation by deactivating proteins encoded by oncogenes. At all stages of the cell cycle, DNA integrity is checked. If damage has occurred and DNA repair is not possible, the cell normally undergoes apoptosis and is eliminated. Thus, apoptosis is a key mechanism for removing cells with DNA damage and which are therefore potentially precancerous. Cancer results from uncontrolled cell proliferation, usually due to gene mutations causing abnormalities in the protein regulators of the cell cycle. Cancerous cells have a decreased ability to undergo apoptosis [4]. 

The process of bronchial carcinogenesis is characterized by accumulated genetic abnormalities which ultimately lead to malignant transformation of bronchial epithelial cells, followed by invasion and metastasis. One of the most common and consistent of these genetic lesions is inactivation of the p53 tumor suppressor gene by mutation or deletion [34]. The frequency of p53 alterations in lung cancer is highest in those subtypes of bronchial carcinomas that are most consistently associated with smoking, especially small cell carcinoma and epidermoid carcinomas. An understanding of the role of p53 in human lung cancer may lead to more rational targeted approaches for treating this disease.

Association of p53 codon 72 polymorphism with lung cancer risk has been studied by several groups although with inconsistent results. The pro allele was found to be in excess in patients with adenocarcinoma in an American study. A study carried out in Japan showed that a significant association of the Pro allele with the small cell carcinoma group, but not in adenocarcinoma [35]. In our study, Pro BstUI and MspI (A1) allele of p53 increased the risk in epidermoid carcinoma and proportion of this allele was observed decreased in large cell carcinoma. However, the ratio of BstUI and MspI A2 allele was observed slightly decreased adenocarcinoma and large cell carcinoma. The hypothesis was that individuals who possess a higher number of variant p53 pro alleles would be at higher risk for lung cancer. Yet, our results only agreed with other case-control studies. These above findings imply that the p53 codon 72 polymorphism may play a putative role in the etiopathogenesis of epidermoid carcinoma in Turkey. 

The estimation of p53 haplotypes revealed an interesting phenomenon. Consequently, the most common (wild-type) haplotype combination is A2-A2, that is, the p53 BstUI A2 allele (or Arg allele) linked to presence of the MspI restriction site. The next haplotype in frequency (A1-A2) differs from the “wild type” in only one mutation, and the third haplotype in frequency (A1-A1) contains two mutational events with respect to the wild type [27]. The haplotype carrying BstUI A2 allele and MspI A1 allele, which was found in only 1.2% among the Swedish controls and in none of Swedish lung cancer patients [36], had a frequency of 5.4% in cancer patients and null among healthy controls in our study. The haplotype carrying the BstUI A2 allele and MspI A1 allele was not found in healthy controls in contrast to the presence of this haplotype in Slovak lung cancer patients (5.4%) [6]. Our results only agreed with those of Birgander et al. when lung cancer patients were compared with healthy controls concerning to BstUI polymorphism [36]. In our study, the haplotype carrying the BstUI A2 allele and MspI A1 allele was not found in healthy controls and schizophrenia in contrast to the presence of this haplotype in Turkish lung cancer patients (12%). It is interesting in this context that there exist the ethnic differences in cancer susceptibility modulated by p53 polymorphic sites. This suggestion is supported by other studies of the codon 72 polymorphism (BstUI), which have revealed striking ethnic differences. The haplotype carrying the The BstUI A1-MspI A2 haplotype was not found in healthy controls and schizophrenia. In addition, the estimated frequency of the BstUI A2-MspI A2 haplotype of lung cancer patients was higher than that of schizophrenia patients. Thus, it could be suggested that the MspI A2 allele, by itself or perhaps more important in the form of the BstUI A1-MspI A2 haplotype, is a marker ofsusceptibility of lung cancer.

However, the significant differences in the genotype and allele frequencies of the MspI polymorphism in the p53 gene between the schizophrenia and the lung cancer patient groups suggest that the p53 polymorphisms could be a marker for lower susceptibility to lung cancer genetically in schizophrenia patients. Furthermore, our results imply that genetic predisposition towards schizophrenia may offer reduced vulnerability to lung cancer. However, further studies involving larger, better age-matched sample populations would be necessary for more concrete results in order to confirm the our results. Moreover, it is necessary to investigate more SNPs in the p53 gene or other genes related to apoptosis for better understanding of the relationship between schizophrenia and cancer.

## Figures and Tables

**Table 1:** General characteristics of the control group, lung cancer patients, and schizophrenia patients.

Characteristic	Lung cancer patients	Schizophrenia patients	Control group
Males *n* (%)	90	54	59
Females *n* (%)	10	46	41
Age, mean (SD), years	60.7 ± 10.8	37.2 ± 10.0	50.3 ± 17.4

Smoking status	*n*	*n*	*n*

Smokers	88	94	43
Nonsmokers	12	6	57

Alcohol consumption	*n*	*n*	*n*

Drinking	34	30	12
Nonalcoholic	66	70	88

**Table 2:** BstUI and MspI polymorphisms of the p53 gene genotype frequencies in Turkish schizophrenia and lung cancer patients and control groups.

	Genotype distribution							
	A1-A1		A2-A2		A1-A2			
BstUI	*n*	%	*n*	%	*n*	%	*X* ^2^	*P*
Schizophrenia (*n* = 100)	38	38.0	43	43.0	19	19.0	9.620	<.01
Lung cancer (*n* = 100)	5	5.0	87	87.0	8	8.0	129.740	<.001
Controls (*n* = 100)	22	22.0	33	33.0	45	45.0	7.940	<.05

MspI								

Schizophrenia (*n* = 100)	0	0.0	46	46.0	54	54.0	.640	.424
Lung cancer (*n* = 100)	13	13.0	43	43.0	44	44.0	18.620	<.001
Controls (*n* = 100)	0	0.0	33	33.0	67	67.0	11.660	<.001

^a^Differences in genotype and allele frequencies; *P* < .01 from Fisher's exact test.

**Table 3:** BstUI and MspI polymorphisms of the p53 gene allele frequencies in Turkish schizophrenia patients, lung cancer patients, and control groups.

		Allele frequency			
		A1	A2		
BstUI	Schizophrenia (*n* = 100)	0.48	0.53	**32.092**	*P* < .001
	Lung cancer (*n* = 100)	0.09	0.91		
	Controls (*n* = 100)	0.45	0.56		

MspI	Schizophrenia (*n* = 100)	0.27	0.73	**27.178**	*P* < .001
	Lung cancer (*n* = 100)	0.35	0.65		
	Controls (*n* = 100)	0.34	0.67		

^a^Differences in genotype and allele frequencies; *P* < .01 from Fisher's exact test.

**Table:** (a) Risk of lung cancer from BstUI genotype with reference to histological types.

Histological types	p53-BstUI type							
	A1/A1		A1/A2		A2/A2		Total	
	*n*	%	*n*	%	*n*	%	*n*	%
Large cell carcinoma	1	20.0	2	28.6	6	8.1	9	10.5
Small cell carcinoma.	1	20.0	2	28.6	26	35.1	29	33.7
Epidermoid carcinoma	1	20.0	3	42.9	28	37.8	32	37.2
Adenocarcinoma	2	40.0	0	0.0	14	18.9	16	18.6

Total	5	100.0	7	100.0	74	100.0	86	100.0

*X* ^2^: 6.400, df:6, *P* = .380.

**Table:** (b) Risk of lung cancer from MspI genotype with reference to histological types.

Histological types	p53-MspI type							
	A1/A1		A1/A2		A2/A2		Total	
	*n*	%	*n*	%	*n*	%	*n*	%
Large cell carcinoma	1	12.5	4	10.8	4	9.8	9	10.5
Small cell carcinoma	2	25.0	12	32.4	15	36.6	29	33.7
Epidermoid carcinoma	4	50.0	13	35.1	15	36.6	32	37.2
Adenocarcinoma	1	12.5	8	21.6	7	17.1	16	18.6

Total	8	100.0	37	100.0	41	100.0	86	100.0

*X* ^2^: 1.146, df:6, *P* = .979.

**Table 5:** The differences of allele frequencies estimated according to histological types of lung cancer patients.

Allele BstUI		A1	A2
Group	Histological types		

Lung cancer	Large cell carcinoma	34.3	22.4
	Small cell carcinoma	34.3	49.4
	Epidermoid carcinoma	41.5	59.2
	Adenocarcinoma	40.0	18.9

Allele MspI			

Lung cancer	Large cell carcinoma	17.9	15.2
	Small cell carcinoma	41.2	52.8
	Epidermoid carcinoma	67.5	54.1
	Adenocarcinoma	23.3	27.9

**Table 6:** Estimated haplotype frequencies of the BstUI and MspI polymorphisms of the p53 gene.

	Estimated haplotype frequency									
	A1-A1		A2-A2		A1-A2		A2-A1			
Haplotype BstUI-MspI	*n*	%	*n*	%	*n*	%	*n*	%	*X* ^2^	*P*
Schizophrenia	0	**0.0**	61	**61.0**	39	**39.0**	0	**0.0**	4.840	<.05
Lung cancer	0	**0.0**	82	**82.0**	6	**6.0**	12	**12.0**	107.120	<.001
Control	0	**0.0**	79	**79.0**	21	**21.0**	0	**0.0**	33.960	<.001

^a^Significant pairwise association and linkage disequilibrium between the two polymorphisms; *P* < .001.

**Table 7:** Estimated pairwise haplotype frequencies between the BstUI and MspI polymorphisms in lung cancer patients and controls.

		Estimated haplotype frequency										
		A1-A1		A2-A2		A1-A2		A2-A1		Total		
Haplotype		*n*	%	*n*	%		%	*n*	%	*n*	*X* ^2^	*P*
Schizophrenia	Male	0	0.0	33	61.1	21	38.9	0	0.0	54	.339	.844
	Female	0	0.0	28	60.9	18	39.1	0	0.0	46		

Lung cancer	Male	0	0.0	74	82.2	5	5.6	11	12.2	90	.001	.98
	Female	0	0.0	8	80.0	1	10.0	1	10.0	10		

Control	Male	0	0.0	48	81.4	11	18.6	0	0.0	59	.481	.488
	Female	0	0.0	31	75.6	10	24.4	0	0.0	41		

^a^ Cases versus control.
